# A call to implement preclinical study registration in animal ethics review

**DOI:** 10.1371/journal.pbio.3002293

**Published:** 2023-10-05

**Authors:** Matthew S. Jeffers, Aileen MacLellan, Marc T. Avey, Julia ML Menon, Janet Sunohara-Neilson, Dean A. Fergusson, Manoj M. Lalu

**Affiliations:** 1 Clinical Epidemiology Program, Ottawa Hospital Research Institute, Ottawa, Canada; 2 School of Epidemiology and Public Health, University of Ottawa, Ottawa, Canada; 3 Department of Integrative Biology, University of Guelph, Guelph, Canada; 4 Canadian Council on Animal Care, Ottawa, Canada; 5 Preclinicaltrials.eu, Netherlands Heart Institute, Utrecht, the Netherlands; 6 Animal Care and Veterinary Services, Western University, London, Canada; 7 Department of Medicine, University of Ottawa, Ottawa, Canada

## Abstract

Protocol registration is required in clinical trials. Registration of animal studies could improve research transparency and reduce redundancy, yet uptake has been minimal. Integrating study registration into institutional approval of animal use protocols is a promising approach to increase uptake.

## Abstract 2

Prospective registration of animal studies could improve research transparency and reduce redundancy. In this Perspective, the authors argue that integrating registration as part of animal ethics review could increase uptake of this practice.

Interventions that appear effective in preclinical animal studies often demonstrate poor success when tested in human clinical trials, which has led to a “translation crisis” in basic science [1]. This is partially due to selective reporting of the most promising study outcomes [2], the “file drawer” effect where negative findings never get published, and failures to account for excluded animals during analysis [3]. These biases result in overestimation of treatment effect sizes in the preclinical literature [4]. Preclinical study registration, the prospective posting of study protocols on publicly accessible registries, offers a way to mitigate these biases. Yet to date, this practice has seen little uptake [5].

In contrast, over the last 2 decades, a series of policy changes and legal requirements from regulatory bodies sharply increased registration of human clinical trials [6]. The US Food and Drug Administration set legal mandates for creation of clinicaltrials.gov and trial registration; the International Committee of Medical Journal Editors made registration a prerequisite for publication; and the US National Institutes of Health began requiring registration of all trials receiving funding. This promoted global adoption of clinical trial registration and mandatory proof of registration for ethical approval of clinical trials. Together, this provides evidence that external organizations can influence registration decisions and promote uptake.

For animal researchers, similar resources exist, with 2 registries specifically for protocol submission (Preclinicaltrials.eu, Animal Study Registry) and other general platforms available for documenting study materials (e.g., Open Science Framework). These publicly accessible databases aim to provide an overview of all animal studies (including those that are ongoing and work that may remain unpublished) to increase transparency and reduce unnecessary duplication of experiments. Unfortunately, usage to date has been low, with only a few hundred entries across each registry [7]. As a potential solution, we propose integrating preclinical registration within the animal use protocol (AUP) review process. This could mirror the successful implementation observed with clinical trials and ultimately increase uptake.

Drawing on previous analyses of reporting in animal experiments [8], views on animal study registries [9], and discussions among the investigative team (comprising preclinical and clinical researchers, veterinarians, members of international preclinical registries, national animal ethics governing bodies, and institutional animal ethics boards), we identified major facilitators and barriers to integrating study registration with AUP review [10]. In Canada and other jurisdictions (e.g., United States, European Union, United Kingdom), oversight bodies such as the Canadian Council on Animal Care empower animal ethics committees to ensure animal users complete AUP forms that detail and justify proposed research. These committees are aware and supportive of the principles and benefits of registration practices, offering a major driver for implementing preclinical study registration at the level of AUP review. Moreover, animal ethics committees also have the infrastructure and regulatory power to institute local requirements for preclinical study registration, and animal users already report much of the information required by registries in their AUPs. Therefore, harmonization of the AUP and animal registry requirements could minimize administrative burden on researchers and facilitate use of animal study registries.

However, a major barrier to implementing preclinical study registration into AUP review is a perceived lack of research community support [9]. Concerns that registration has potential to cause harm to researchers by increasing regulatory burden and risking intellectual property loss (i.e., having experimental plans “scooped”), as well as limited time and resources available to adapt review processes, contributes to hesitancy. These perceptions must be addressed to support the collaborative relationship between scientists and animal ethics committees while promoting implementation of study registration.

Ensuring successful implementation of this approach will require an in-depth assessment of current practices at a variety of institutions. Doing so is essential for gauging compatibility with preclinical registration requirements and identifying areas of opportunity and institutional readiness for integration of preclinical registration and animal ethics review (Table 1). For example, hypothesis-testing research would be an ideal target for initial implementation. In the biomedical sphere, these translationally oriented “confirmatory” studies often feature the predefined hypotheses, statistical analysis plans, and design elements (e.g., randomization/blinding) that most closely resemble human clinical trials where registration is already widely practiced [11,12]. However, preclinical study registration may also be readily adopted in other areas (e.g., exploratory or basic research), since existing registries include a flexible registration process that facilitate descriptions of diverse protocols (e.g., researchers conducting exploratory work or pilot studies can justify the absence of a priori sample size calculations; basic researchers can include details of experimental designs). Still, to build research community approval for study registration, working groups of preclinical researchers and other institutional stakeholders (e.g., committee members, veterinarians) should be established to liaise with organizations that have successfully adopted study registration into their AUP review processes. This will allow institutions to learn from past successes/challenges, build support, and reach consensus on how study registration practices can be successfully implemented at a local level (Table 1).

**Table 1:** Potential intervention strategies to overcome barriers to integration of AUP review and study registration.

Intervention component	Setting the stage	Barrier #1: Social influences	Barrier #2: Environmental context and resources
**Aims:** What will the intervention achieve and for whom?	• Assess current practices, areas of opportunity, and institutional readiness for implementing registration into ethics review	• Obtain support and list of requested changes to animal use form from local preclinical research community	• Change AUPs of preclinical studies intended for clinical translation to align with study registration requirements
**Ingredients:** What comprises the intervention?	• Systematic review of institutional animal use protocol forms and processes • Interviews with members of institutional animal ethics committees	• Establishing institutional working group of affected preclinical researchers and other stakeholders (veterinarians, etc.) • Meet to discuss strategies and research initiatives to improve rigor and translation of preclinical health research	• Work with animal ethics committee to guide changes to AUP forms based on research community priorities • Provide educational materials/workshops to reduce institutional burden of educating researchers on new registration procedures
**Mechanism:** How will intervention work?	• Prepare institutions to be active participants in implementation of registration • Conduct local needs assessments • Audit and provide feedback on current local AUP review processes • Develop a formal implementation blueprint for local institutions	• Advisory boards and workgroups • Creating a learning collaborative • Implementation advisor (study registry) • Identification of early adopters that have implemented registration processes • Visit sites where registration has been implemented • Obtain formal institutional commitments	• Provision of technical assistance • Facilitation of process changes • Changes to record systems • Developing quality monitoring systems • Identification and preparation of champions (relevant study coordinators) • Developing academic partnerships • Creating a learning collaborative
**Delivery:** How will the intervention be delivered?	• Published literature review and qualitative assessments of animal ethics committee views • Reports to institutional stakeholders on current practices and areas of opportunity for harmonization/improvement	• Regular meetings with institutional working group • Collaboration with study registry and/or other institutions that have implemented registration practices	• Regular follow-up with animal ethics committees to monitor implementation • Collaboration with study registry (as needed by animal ethics committee) • Delivery of training workshops to educate researchers • Guidance documents for new registration procedures

Furthermore, assisting animal ethics committees with adaptations to AUPs will promote implementation and minimize researcher burden through harmonization of ethics review and study registration forms. Practical social support should be provided in the form of technical assistance by animal study registries for interactive problem-solving and support with implementation challenges (Table 1). Dissemination of educational materials created by animal study registries, as well as educational workshops for researchers affected by registration requirements, will be helpful to prepare the community and mitigate concerns about changes to the ethics process. Having local champions of preclinical registration provide training on behalf of the animal ethics committee could facilitate adoption, strengthen relationships between researchers and animal ethics committees, and alleviate barriers caused by limited resources and the novelty of preclinical study registration.

Throughout the implementation process, all affected groups (researchers, ethics committees, etc.) should be engaged and consulted to ensure that changes to the ethics review and registration processes are perceived as feasible, acceptable, and working as intended. Thus, the efficacy of this approach could be evaluated through working groups, surveys, and semi-structured interviews to collect data that would help guide improvement of future implementation initiatives. Additionally, the systems impact of implementing these practices should be monitored through before-after assessments of registration numbers at each preclinical study registry, as well as the ratio of approved AUPs (and thus registered protocols) to published experiments as a measure of publication bias.

Overall, integration of preclinical study registration into ethics review facilitates uptake of this practice, making it a routine element of animal research and potentially enabling seamless transmission of approved AUPs from the ethics committee to the study registry. Developing partnerships between local researchers, animal ethics committees, and animal study registries will promote feasibility and sustainability of this practice. Critically, this approach will help researchers and animal ethics committees alike by minimizing the administrative burden of having separate AUP and registration processes, promoting detailed reporting, providing transparency in whether published research was carried out as initially approved, and reducing redundancy in animal studies.

We suggest a pragmatic approach to implementation of study registration into institutional animal ethics review through assessment of the current landscape, building community support, and connecting research, ethics, and registry stakeholders. Implementing preclinical study registration is a critical step in improving the transparency of preclinical research intended to inform clinical trials. As a research community, we must undertake these steps in order to reduce failed translation due to the negative impacts of selective outcome reporting, publication bias, and overestimation of preclinical treatment efficacy [2–4].
